# Three-Dimensional Free Vibration Analyses of Preloaded Cracked Plates of Functionally Graded Materials via the MLS-Ritz Method

**DOI:** 10.3390/ma14247712

**Published:** 2021-12-14

**Authors:** Chiung-Shiann Huang, Hao-Ting Lee, Pin-Yu Li, Ming-Ju Chang

**Affiliations:** 1 Department of Civil Engineering, National Yang Ming Chiao Tung University, 1001 Ta-Hsueh Rd., Hsinchu 30010, Taiwan; god11011@gmail.com (H.-T.L.); kenlee0609@yahoo.com.tw (P.-Y.L.); 2 Intelligent Autonomous Guided Vehicle RD Center, Coretronic Corp., Hsinchu 30010, Taiwan; mjchang0123@gmail.com

**Keywords:** vibrations, MLS-Ritz method, enriched basis functions, cracked FGM plates, three-dimensional elasticity

## Abstract

In this study, the moving least squares (MLS)-Ritz method, which involves combining the Ritz method with admissible functions established using the MLS approach, was used to predict the vibration frequencies of cracked functionally graded material (FGM) plates under static loading on the basis of the three-dimensional elasticity theory. Sets of crack functions are proposed to enrich a set of polynomial functions for constructing admissible functions that represent displacement and slope discontinuities across a crack and appropriate stress singularity behaviors near a crack front. These crack functions enhance the Ritz method in terms of its ability to identify a crack in a plate. Convergence studies of frequencies and comparisons with published results were conducted to demonstrate the correctness and accuracy of the proposed solutions. The proposed approach was also employed for accurately determining the frequencies of cantilevered and simply supported side-cracked rectangular FGM plates and cantilevered internally cracked skewed rhombic FGM plates under uniaxial normal traction. Moreover, the effects of the volume fractions of the FGM constituents, crack configurations, and traction magnitudes on the vibration frequencies of cracked FGM plates were investigated.

## 1. Introduction

The material properties of functionally graded materials (FGMs) exhibit inhomogeneity. By incorporating gradual changes in the compositions of the constituent gradients along one direction, FGMs can be designed to achieve a desired goal, such as high resistance to temperature gradients and corrosion, high toughness, and high strength. In contrast to laminated composite materials, FGMs do not exhibit stress concentration at the interface of two adjacent layers. Consequently, FGMs are crucial composite materials that are used in diverse engineering applications.

Plates are widely used structural components. Several studies [1,2,3,4] have reviewed the literature on static and dynamic analyses of FGM plates based on various plate theories and the three-dimensional elasticity theory. Most of the relevant studies in the literature have analyzed intact flat plates. Analytical solutions based on plate theories and the three-dimensional elasticity theory have been proposed for the vibration of rectangular plates with two simply supported opposite edges and four simply supported faces, respectively [5,6,7,8,9,10,11]. Moreover, solutions for the vibration of rectangular plates under different boundary conditions have been reported using various numerical approaches, such as the Ritz method [12,13,14], differential quadrature method [15,16,17], mesh-free method [18,19,20], and finite-element method (FEM) [21,22,23].

Cracks are initiated in a material due to material flaws, cyclic loading, or stress concentration. Under resonance, such cracks propagate rapidly and cause a plate to malfunction. Consequently, determining the vibration characteristics of a cracked plate is crucial. Numerous studies have investigated the vibrations of cracked homogeneous and isotropic plates without static loading. On the basis of the classical plate theory (CPT), these studies have used analytical methods [24,25], the integral equation approach [26,27], the Ritz method [28,29,30,31], the generalized differential quadrature method [32,33], Galerkin’s method [34], the mesh-free approach [35], and the FEM [36,37] to investigate the aforementioned vibrations. Based on the first-order shear deformation plate theory (FSDT), studies have developed various approaches, such as the Ritz method [38,39], mesh-free method [40], FEM [41,42], and extended FEM (XFEM) [43,44], while Singh et al. [45] used an extended isogeometric approach based on a higher-order shear deformation plate theory.

The inhomogeneity of FGM plates induces coupling among their three displacement components and complicates their vibration analyses. Vibration analyses of cracked FGM plates have been carried out using various plate theories and the three-dimensional elasticity theory along with different numerical approaches. Based on FSDT, Natarajan et al. [46] investigated the vibration of cracked FGM plates by using an XFEM with four-node quadrilateral plate-bending elements, while Nguyen-Thoi et al. [47] employed an XFEM with cell-based smoothed three-node elements. Fantuzzi et al. [48] presented a generalized differential quadrature finite-element approach to analyze the vibration of rectangular FGM plates with internal cracks. Yin et al. [49] and Zhang et al. [50] combined extended isogeometric analysis with nonuniform rational B-spline basis functions to analyze the aforementioned vibration, while Khalafia and Fazilati [51] investigated vibrations of plates containing embedded curved cracks. To determine the dynamic characteristics of cracked thick FGM plates based on Reddy’s third-order shear deformation plate theory, Huang et al. [52] and Tran et al. [53] employed the Ritz method and an extended isogeometric approach, respectively. Moreover, Huang et al. [54,55] performed three-dimensional vibration analyses by using the Ritz method. The Ritz solutions proposed in [52,54,55] have continuous admissible functions, including crack functions, with support covering the entire domain of the problem under consideration. Numerical difficulties are encountered before obtaining convergent vibration frequencies when using these solutions. To delay the occurrence of such numerical difficulties for obtaining convergent vibration frequencies, 128-bit precision variables were used in the computer codes developed in the studies via the Ritz method.

Investigating the vibration behaviors of cracked plates under static loading is crucial because a structural plate component is typically subjected to different loadings. Moreover, a crack complicates the distributions of stress components in a loaded plate and substantially increases the complexity of vibration analysis of such a plate under static loading. However, the literature on this topic is limited. Relevant previous studies are based on CPT or FSDT. Petyt [56] employed CPT and a finite element approach to determine the fundamental frequencies of rectangular homogeneous plates with central vertical cracks under uniform tensile loads on the edges parallel to the crack. Vafai et al. [57] reported the fundamental frequencies of side-cracked rectangular plates with four simply supported edges subjected to uniform uniaxial in-plane loading by solving an integro-differential equation derived from CPT. The side cracks considered by Vafai et al. [57] were parallel to the applied loading, which indicates that the stress resultants were uniformly distributed in the plates. Zeng et al. [58] and Huang et al. [59] accurately computed the natural frequencies and vibration mode shapes of cracked thin homogeneous plates under in-plane loading by using the MLS-Ritz method. To analyze the vibration of FGM plates with central internal cracks and subjected to in-plane thermal loading based on FSDT, Natarajan et al. [60] employed an FEM with eight-node shear flexible elements, while Rahimabadi et al. [61] utilized an XFEM with eight-node shear flexible elements and enriched shear flexible four-node quadrilateral elements.

The main purpose of this study was to propose a numerical solution for free vibration of a cracked FGM plate with static loading using the three-dimensional elasticity theory along with the MLS-Ritz method. Instead of using a plate theory, which represents a simplified form of the three-dimensional elasticity theory under various assumptions, we used the three-dimensional elasticity theory to investigate the free vibration of a cracked FGM plate subjected to in-plane static loading. The solution presented in this paper comprises two main stages. In the first stage, a cracked FGM plate subjected to in-plane static loading is analyzed, and the distributions of six stress components, which are referred to as initial stresses in this paper, are determined. In the second stage, the free vibration of the cracked plate is analyzed by considering the effects of the initial stresses. Unlike plate theories, which consider only the in-plane initial stress resultants, the effects of six stress components on the vibration frequencies of a plate were considered in this study.

We adopted the well-known Ritz method to perform free vibration analyses of cracked and loaded FGM plates with varying material properties along their thickness direction (*z*). In both static and vibration analyses, the admissible functions of the three displacement components comprise regular polynomials in the thickness direction (*z*) multiplied by admissible (*x*, *y*) functions constructed using the MLS technique [62]. The set of basis functions used in the MLS technique contains regular polynomial functions and crack functions, which represent not only the correct singularity orders of stresses at a crack front but also discontinuous displacement across a crack. The validity of proposed approach was confirmed by conducting comprehensive convergence studies and comparing the obtained results with the published vibration frequencies of cracked FGM plates without loading and the vibration frequencies of cracked and loaded homogeneous plates obtained using the ANSYS commercial finite-element software package (mechanical/Ansys 19.0, ANSYS, Inc., Canonsburg, PA, USA). An extensive amount of vibration frequencies was tabulated for simply supported and cantilevered square, rectangular, and skewed rhombic FGM plates with side cracks and internal cracks under uniaxial compression and tension to expand the database in the literature. Moreover, we investigated the natural frequencies of cracked plates with different material properties, loading magnitudes, plate geometries (side–side ratios, side–thickness ratios, and skew angles), and crack configurations (crack lengths, crack locations, and crack orientations). These results can serve as benchmark values for evaluating the accuracy of other numerical methods and various plate theories.

## 2. Mathematical Formulation

**Figure 1:**
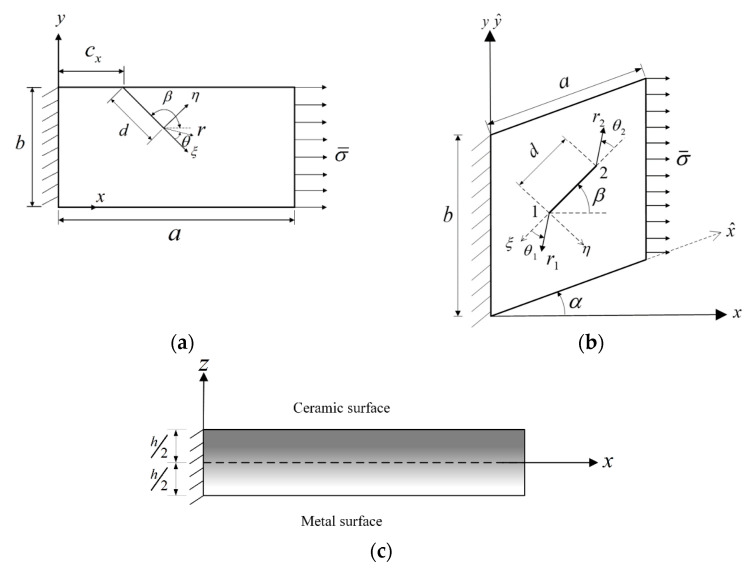
A preloaded cantilevered cracked FGM plate with coordinate systems: (**a**) Top view of a side-cracked plate, (**b**) Top view of an internally cracked plate, (**c**) Side view.

The dimensions of a cracked FGM plate and the geometric parameters of the crack configuration are depicted in Figure 1, which also displays the various coordinate systems used in this study. The FGM under consideration is composed of ceramic and metallic constituents. The ceramic surface is located at *z* = *h*/2, and the metallic surface is located at *z* = −*h*/2, where *h* is the plate thickness.

The effective material properties of FGMs mainly depend on the size, aspect ratio, and spatial distribution of the particles embedded in the matrix. In this study, we estimated the effective material properties by using the rule of mixture. Variations in material properties in the thickness direction (*z*) are described using the power law presented in Equation (1) [63,64], which has been widely used in the literature.

(1) $$P(z)=(P_{c}-P_{m})V(z)+P_{c}\;\mathrm{and}\;V(z)={\left(\frac{z}{h}+\frac{1}{2}\right)}^{\bar{m}}$$

where $P_{c}$ and $P_{m}$ are the material properties (i.e., the elastic modulus *E*, Poisson’s ratio ν, and the mass density ρ) of the ceramic and metallic constituents, respectively; and $\bar{m}$ is the power-law index governing the material variation profile along the thickness direction. The FGM plates considered herein are made of aluminum (Al) and ceramic (alumina (Al_2_O_3_)) of *E* = 70 and 380 GPa, ρ = 2702 and 3800 kg/m^3^**,** respectively, and ν = 0.3 [8,54,65]

### 2.1. Static Stress Analyses

Static stress analyses were conducted to determine the initial stresses inside a cracked FGM under static loading according to the Ritz method and three-dimensional elasticity theory. The total energy functional is expressed as follows:

(2) $$\Pi =\frac{1}{2}{∭}_{V}\sigma^{\left(0\right)}:\;\varepsilon^{\left(0\right)}\;dV-\iint_{S}t\cdot u^{\left(0\right)}dA$$

where σ and ε are the Cauchy stress tensor and the infinitesimal strain tensor, respectively; **t** and **u** are the traction and displacement vectors, respectively; the superscript (0) denotes the physical quantities in the static problem; and *S* is the boundary surface with the prescribed traction. Notably, **u** satisfies the displacement-prescribed boundary conditions. By substituting the linear stress–strain and strain–displacement relations into Equation (2), the following equation is obtained:

(3) $$\Pi =\frac{1}{8}{∭}_{V}\tilde{C}\cdot (\nabla u^{\left(0\right)}+{\left(\nabla u^{\left(0\right)}\right)}^{T}):\;(\nabla u^{\left(0\right)}+{\left(\nabla u^{\left(0\right)}\right)}^{T})\;dV-\iint_{S}t\cdot u^{\left(0\right)}dA$$

where $\tilde{C}$ is the fourth-order tensor of material elastic constants, which are functions of *z* for an FGM plate, and ∇ is the del operator.

Let $u={\left(u_{1},u_{2},u_{3}\right)}^{T}$, which are the displacement components along the *x*-direction, *y*-direction, and *z*-direction, respectively. For easily introducing asymptotic fields of displacements at a crack front into the constructed solution and accurately describing the stress singular behaviors near the crack front, *u*_1_ and *u*_2_ are expressed in terms of ${\bar{u}}_{1}\;\mathrm{and}\;{\bar{u}}_{2}$, which are the displacement components along the ξ-direction and η-direction, respectively (Figure 1), as follows:

(4) $$\left\{\begin{array}{c} u_{1}\\ u_{2} \end{array}\right\}=\left[\begin{array}{cc} -\cos\beta & \sin\beta \\ -\sin\beta & -\cos\beta \end{array}\right]\left\{\begin{array}{c} {\bar{u}}_{1}\\ {\bar{u}}_{2} \end{array}\right\}$$

where β (Figure 1) is the inclination angle of a crack. The displacement functions ${\bar{u}}_{1},\;{\bar{u}}_{2}$, and $u_{3}$ are expanded using sets of admissible functions $\left\{\Phi_{\gamma j}(x,y,z)|j=1,2,\ldots ;\gamma =1,2\;\mathrm{and}\;3\right\}$ as follows:

(5) $$\begin{array}{l} {\bar{u}}_{\gamma }^{\left(0\right)}=\sum_{j=1}^{Nt}{\bar{A}}_{\gamma j}\Phi_{\gamma j}(x,y,z)=\sum_{i=1}^{Np}\sum_{k=0}^{Nz}u_{\gamma ik}^{\left(0\right)}\phi_{\gamma \;i}(x,y)z^{k}\;(\gamma =1,\;2)\;\\ \mathrm{and}\;u_{3}^{\left(0\right)}=\sum_{j=1}^{Nt}{\bar{A}}_{3j}\Phi_{3j}(x,y,z)=\sum_{i=1}^{Np}\sum_{k=0}^{Nz}u_{3ik}^{\left(0\right)}\phi_{3\;i}(x,y)z^{k} \end{array}$$

where $\phi_{li}(x,y)$ (*l* = 1, 2, 3), which is constructed using the MLS technique, accurately describes the singular stresses at a crack front and highlights the discontinuity of displacement across the crack. The development of $\phi_{li}(x,y)$ is shown below. Because the thickness of the plate considered herein is smaller than its length and width, a set of regular polynomials of *z* is employed in Equation (5) to facilitate independent integration with respect to *z* in the volumetric and area integrations required in Equation (3).

By substituting Equations (4) and (5) into Equation (3) and minimizing the energy functional, we obtain a set of 3×Np×(Nz+1) linear algebraic equations, which are expressed as follows:

(6) $$\left[\begin{array}{ccc} {Κ}^{11} & {Κ}^{12} & {Κ}^{13}\\ & {Κ}^{22} & {Κ}^{23}\\ sym & & {Κ}^{33} \end{array}\right]\left\{\begin{array}{c} {\hat{u}}_{1}{}^{\left(0\right)}\\ {\hat{u}}_{2}{}^{\left(0\right)}\\ {\hat{u}}_{3}{}^{\left(0\right)} \end{array}\right\}=\left\{\begin{array}{c} f^{1}\\ f^{2}\\ f^{3} \end{array}\right\}$$

where ${\hat{u}}_{l}^{\left(0\right)}\;=\;\;{\left(u_{l10}^{\left(0\right)},u_{l20}^{\left(0\right)},u_{l30}^{\left(0\right)},\cdots ,u_{lN_{p}0}^{\left(0\right)},u_{l11}^{\left(0\right)},\cdots ,u_{lN_{p}N_{z}}^{\left(0\right)}\right)}^{T}$, and the expressions for the components of **K**^ij^ and **f**^i^ are given in Appendix A. The static displacements are determined using Equations (4) and (5) after obtaining ${\hat{u}}_{l}^{\left(0\right)}\;$ by solving Equation (6). Then, the initial stresses are calculated using the strain–displacement and stress–strain relationships.

### 2.2. Vibration Analyses

The static deformations considered in the previous section are assumed to be sufficiently small to be neglected when considering the plate geometry. The Ritz method is used to analyze the free vibration of a cracked FGM plate under static loading, and the total energy functional including the work done by the initial stresses is expressed as follows:

(7) $$\Pi =\frac{1}{2}{∭}_{V}\left(\sigma :\varepsilon +\sigma_{}^{\left(0\right)}:(\nabla u\cdot {\left(\nabla u\right)}^{T})\right)\;dV-\frac{\omega^{2}}{2}{∭}_{V}\rho u\cdot udV$$

where u denotes a vector of the vibration amplitudes along the *x*-direction, *y*-direction, and *z*-directions and ω denotes the angular frequency. To simplify the calculation, the admissible functions in Equation (5) are used to linearly expand u in Equation (7). Therefore, the expressions of u are identical to those in Equation (5) without the superscript (0). By minimizing the energy functional presented in Equation (7), we obtain 3×Np×(Nz+1) linear algebraic equations from ∂Π∂ulik=0 (l=1,2 and 3) and form a generalized eigenvalue problem:

(8) $$\left(\left[\begin{array}{ccc} K^{11} & K^{12} & K^{13}\\ & K^{22} & K^{23}\\ sym & & K^{33} \end{array}\right]+\left[\begin{array}{ccc} K_{g}^{11} & 0 & 0\\ 0 & K_{g}^{22} & 0\\ 0 & 0 & K_{g}^{33} \end{array}\right]\right)\left\{\begin{array}{c} {\hat{u}}_{1}\\ {\hat{u}}_{2}\\ {\hat{u}}_{3} \end{array}\right\}=\omega^{2}\left[\begin{array}{ccc} M^{11} & 0 & 0\\ 0 & M^{22} & 0\\ 0 & 0 & M^{33} \end{array}\right]\left\{\begin{array}{c} {\hat{u}}_{1}\\ {\hat{u}}_{2}\\ {\hat{u}}_{3} \end{array}\right\}$$

where ${\hat{u}}_{l}^{}\;=\;\;{\left(u_{l10}^{},u_{l20}^{},u_{l30}^{},\cdots ,u_{lN_{p}0}^{},u_{l11}^{},\cdots ,u_{lN_{p}N_{z}}^{}\right)}^{T}$ and $K_{g}^{ii}$ results from the work done by the initial stresses. The expressions of $K_{g}^{ii}$ and $M_{}^{ii}$ are presented in Appendix A. Since the same admissible functions are used in static stress analysis and vibration analysis, the value of $K^{ij}$ in Equation (8) is the same as that in Equation (6), which reduces the computational time to some extent.

### 2.3. Admissible Functions

Belytschko et al. [66] first proposed the MLS approach to establish shape functions for the element-free Galerkin method. Following such procedure, the shape functions are constructed and expressed as

(9) $$\phi_{lj}(x,y)=\Gamma_{l}^{T}(x,y)B_{lj}(x,y)\;\mathrm{for}\;l=1,\;2\;\mathrm{and}\;3$$

where

(10) $$\Gamma_{l}(x,y)=A_{l}^{-1}(x,y)p_{l}(x,y),$$

(11) $$A_{l}(x,y)=\sum_{n=1}^{Np}\bar{W}(x-x_{n},y-y_{n})\;p_{l}(x_{n},y_{n})\;p_{l}^{T}(x_{n},y_{n}),$$

(12) $$B_{lj}(x,y)=\bar{W}(x-x_{j},y-y_{j},\;d_{s})p_{l}(x_{j},y_{j}),$$

*N_p_* is the total number of nodal points (*x_j_*, *y_j_*); the column vector $p_{l}$ represents a set of basis functions; and $\bar{W}$ denotes a positive-definite weight function with the support size $d_{s}$, which localizes the shape functions. The following weight function is adopted in this study:

(13) $$\bar{W}\left(x-x_{j},y-y_{j},\;d_{s}\right)=\{\begin{array}{cc} \frac{2}{3}-4{\bar{d}}^{2}+4{\bar{d}}^{3} & if\;\bar{d}\leq \frac{1}{2},\\ \frac{4}{3}-4\bar{d}+4{\bar{d}}^{2}-\frac{4}{3}{\bar{d}}^{3} & if\;\frac{1}{2}<\bar{d}\leq 1,\\ 0 & if\;1<\bar{d}, \end{array}$$

where d¯=(x−xj)2+(y−yj)2ds. This weight function and its first derivatives are continuous. The continuities of a shape function and its derivatives depend on the adopted weight function and basis functions.

Equations (3) and (7) contain the first derivatives of the shape functions, which are implicitly evaluated using Equation (14) because the constructed shape functions are not expressed in an explicit form.

(14) $$\phi_{lj,}{}_{\gamma }=\Gamma_{l,\gamma }^{T}B_{lj}+\Gamma_{l}^{T}B_{lj,\gamma },$$

where

(15) $$\;\Gamma_{l,\gamma }=A_{l}^{-1}(p_{l,\gamma }-A_{l,\gamma }\;\Gamma_{l}).$$

The subscript γ refers to the coordinates *x* and *y*, and the subscript comma represents the partial derivative of the variable after the comma.

Polynomial basis functions are typically used to construct shape functions, and the use of polynomial basis functions leads to the shape functions and their derivatives being continuous over the entire problem domain. Such shape functions cannot approximate the discontinuity behaviors of a displacement function across a crack. Consequently, other basis functions are required to describe such discontinuities.

Hartranft and Sih [67] applied the eigenfunction expansion method to develop asymptotic solutions for the stress singularities at the terminus of a crack in an isotropic and homogeneous plate according to the three-dimensional elasticity theory. The asymptotic solutions were expanded by $p_{in}$ (Equation (16)) and $p_{out}$ (Equation (17)) for in-plane and out-of-plane displacements, respectively.

(16) $$p_{in}^{T}(r,\theta )=\left(r^{1/2}\cos(1/2)\theta ,r^{1/2}\cos\left(3/2\right)\theta ,\;r^{1/2}\sin(1/2)\theta ,\;r^{1/2}\sin\left(3/2\right)\theta \right).$$

(17) $$p_{out}^{T}(r,\theta )=\left(r^{3/2}\cos(3\theta /2),\;r^{1/2}\sin(\theta /2)\right).$$

The polar coordinate system (r,θ) is depicted in Figure 1a.

The functions in Equation (16) represent the opening and sliding fracture modes, and the term $r^{1/2}\sin(\theta /2)$ in Equation (17) represents the tearing fracture mode. Because $r^{1/2}\sin(\theta /2)$ is antisymmetric about θ=0, a symmetric function $r^{3/2}\cos(3\theta /2)$, which does not induce stress singularities at r=0 but yields a discontinuous slope across the crack, is included in Equation (17). Notably, the functions with $r^{1/2}$ induce stress singularities as *r* approaches 0, and the sine functions and first derivatives of the cosine functions are discontinuous across the crack (θ=±π). These functions are called side crack basis functions in this paper, and they enable cracks to be recognized when using the Ritz method.

To construct admissible functions ($\phi_{li}(x,y)$) for a side-cracked plate, the present approach proposes $p_{l}$ for Equation (10) as

(18) $$p_{l}^{T}=g_{l}(x,y)(p_{p}^{T},p_{cl}^{T})\;$$

where $p_{p}^{T}=(1,x,y,x^{2},xy,y^{2})$, $p_{cl}^{}=p_{in}$ for *l* = 1 and 2, $p_{c3}^{}=p_{out}$, and $g_{l}(x,y)$ ensures that the geometry boundary conditions are satisfied. When the geometry of the plate is simple, $g_{l}(x,y)$ can easily be found. If $g_{l}(x,y)$ is difficult to construct, one can modify total energy functionals in Equations (2) and (7) by applying Lagrange multiplier technique [66] or penalty method [68] to take care of the problem caused by the used shape functions that do not satisfy the geometry boundary conditions.

For an internally cracked plate, the two sets of functions in Equations (16) and (17) are inappropriate not only because such a plate has two crack fronts but also because these functions do not correctly describe the internal crack. Therefore, two sets of internal crack functions are proposed in Equations (19) and (20) to replace those presented in Equations (16) and (17), respectively.

(19) $${\bar{p}}_{in}^{T}(r,\theta )=(r_{2}^{1/2}\sin^{2}(\theta_{2}/2)p_{in}^{T}(r_{1},\theta_{1}),\;r_{1}^{1/2}\sin^{2}(\theta_{1}/2)p_{in}^{T}(r_{2},\theta_{2}))\;,$$

And

(20) $$\begin{array}{ll} {\bar{p}}_{out}^{T}= & (r_{2}^{1/2}\sin^{2}(\theta_{2}/2)\;r_{1}^{1/2}\sin(\theta_{1}/2),\;r_{2}^{3/2}\sin^{2}(\theta_{2}/2)\;r_{1}^{3/2}\cos(\theta_{1}/2),\\ & r_{1}^{1/2}\sin^{2}(\theta_{1}/2)r_{2}^{1/2}\sin(\theta_{2}{/2),\;r}_{1}^{3/2}\sin^{2}(\theta_{1}/2)\;r_{2}^{3/2}\cos(\theta_{2}/2)\;),\; \end{array}$$

where two polar coordinate systems $\left(r_{1},\;\theta_{1}\right)$ and $\left(r_{2},\;\theta_{2}\right)$ are defined in Figure 1b. The effects of $r_{l}^{1/2}\sin^{2}(\theta_{l}/2)$ are explained in the following text. For example, $p_{in}^{T}(r_{1},\theta_{1})$ yields a shape function and its first derivatives discontinuous at $\theta_{2}=0$ (Figure 1b), which are undesirable because the internal crack does not exist at that location. The function $r_{2}^{1/2}\sin^{2}(\theta_{2}/2)$ remedies such problem without changing the symmetry of each function in $p_{in}^{T}(r_{1},\theta_{1})$ with respect to $\theta_{1}$, and $r_{2}^{1/2}\sin^{2}(\theta_{2}/2)p_{in}^{T}(r_{1},\theta_{1})$ yields correct singular orders of stresses at $r_{2}=0$ and $r_{1}=0$.

### 2.4. Boundary Conditions

The top (*z* = *h*/2) and bottom (*z* = −*h*/2) surfaces of the plates investigated in this study are stress-free. Moreover, two combinations of boundary conditions, namely CFFF and SSSS, where C, F, and S indicate the clamped, free, and simply supported boundary conditions, respectively, are applied to the four side faces. Notably, only out-of-plane displacement is constrained on a simply supported face. For a cantilevered (CFFF) plate, the face with *x* = 0 is clamped, and uniform normal traction with a magnitude of $\bar{\sigma }$ is specified for the face with *x* (or $\hat{x}$) = *a* (Figure 1). For an SSSS plate, uniform normal traction with a magnitude of $\bar{\sigma }$ is specified for the faces with *x* = 0 and *x* = *a*. To satisfy the essential boundary conditions, $g_{l}(x,y)=x$ in Equation (18) is used for CFFF plates, while *g*_3_(*x*,*y*) = *xy*(*a* − *x*)(*b* − *y*) and $g_{1}(x,y)=g_{2}(x,y)=1$ are adopted for SSSS rectangular plates.

## 3. Convergence and Comparison Studies

**Figure 2:**
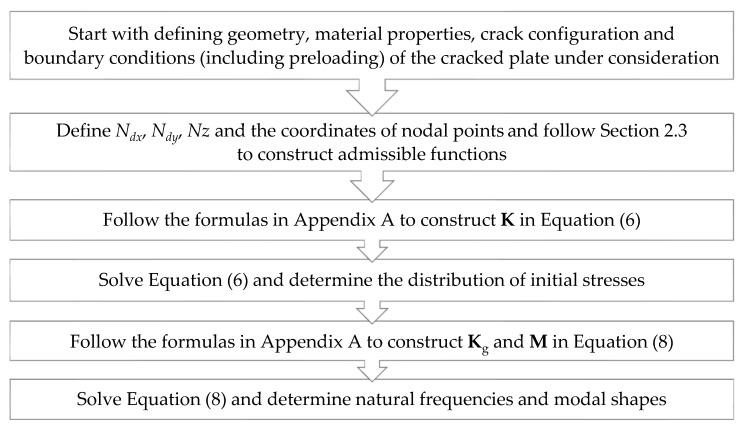
The flow chart for the proposed solution.

After the needed equations have been formulated for developing the proposed solutions for free vibrations of cracked and statically loaded FGM plates, the flow chart in Figure 2 simply describes the procedure of constructing the proposed solutions. Equations (9)–(12) indicate that the nodal points inside the problem domain must be assigned to construct the shape functions $\phi_{lj}(x,y)$. We use uniformly distributed nodal points in the domain [0.01a, 0.99a]×[0.01b, 0.99b] with Δx (or $\Delta \hat{x}$) = 0.98*a*/*N_dx_* and $\Delta y\;(\mathrm{or}\;\Delta \hat{y})=$ 0.98*b*/*N_dy_*, where (*N_dx_* +1) and (*N_dy_* +1) denote the numbers of nodal points in x (or $\hat{x}$) and y (or $\hat{y}$) directions, respectively. The total number of nodal points is $N_{p}=(N_{dx}+1)\times (N_{dy}+1)-N_{crack}$, where $N_{crack}$ is the number of nodal points on a crack. For simplicity, let *N_dx_* = *N_dy_* = *N_d_* for a square or skewed rhombic plate.

The accuracy of the solutions obtained using the MLS-Ritz method mainly depends on the number of admissible functions. According to Table 1 and Table 2, the first five nondimensional vibration frequencies ($\Omega =\omega \;(b^{2}/h)\sqrt{\rho_{c}E_{c}}$) of cracked rectangular plates with CFFF boundary conditions converge with the number of admissible functions $N_{t}=N_{p}\times (N_{z}+1)$, where *N_z_* refers to the highest order of *z* in the admissible functions. The results obtained in this study were compared with those of existing studies and those obtained using a commercial finite-element software package to validate the accuracy of the proposed solution.

Table 1 summarizes the convergence of Ω for an FGM rectangular plate ($\bar{m}$ = 0.2, *a*/*b* = 2, and *h*/*b* = 0.1) with a vertical side crack having length (*d*/*b*) of 0.5 at $c_{x}/a=$ 0.5 with and without the uniform normal compressive traction $\bar{\sigma }$ at *x* = *a*. The nondimensional parameter $\beta_{p}=\bar{\sigma }/{\bar{\sigma }}_{cbs}$ is used to indicate the magnitude of $\bar{\sigma }$, where ${\bar{\sigma }}_{cbs}$ is the critical buckling compressive stress for the plate under consideration without a crack. The parameter ${\bar{\sigma }}_{cbs}$ represents the smallest $\bar{\sigma }$ value that yields zero vibration frequency. Notably, ${\bar{\sigma }}_{cbs}$ was determined by the present approach using polynomial basis functions for $\phi_{lj}(x,y)$. Table 3 lists the nondimensional critical buckling loads ${\bar{N}}_{cbs}={\bar{\sigma }}_{cbs}b^{2}h/(\pi^{2}D_{m})$ needed in the present study, where *D_m_* = *E_m_h*^3^/(12(1 − $\nu_{m}^{2}$)). The results in Table 1 were obtained using different numbers of admissible functions (*N*_z_ = 2, 3, and 4; $N_{dx}$ × $N_{dy}$ = 25 × 25, 40 × 20, 30 × 30, 50 × 25) and setting $(d_{m}{}_{\_in}/b,\;d_{m}{}_{\_out}/b)=$ (0.5, 0.8), where $d_{m}{}_{\_in}$ and $d_{m}{}_{\_out}$ denote the supports of the weight function for in-plane and out-of-plane displacements, respectively. As presented in Table 1, the frequencies converge gradually as the number of admissible functions increases (i.e., increase in *N*_z_, $N_{dx}$, and $N_{dy}$). Notably, the in-plane displacements are typically coupled with out-of-plane displacement in the vibration of an FGM plate. The results marked with “*” refer to modes with dominant in-plane displacements, whereas the other modes have dominant out-of-plane displacements. Increasing *N*_z_ = 3 to *N*_z_ = 4 improves the results only at the fourth significant figure at most. By using *N*_z_ = 4 and $N_{dx}$ × $N_{dy}$ = 50 × 25, convergence is achieved at three significant figures.

Table 1 also lists the frequencies determined by Wang [69] for an FGM plate without static loading. As shown in Huang et al. [55], these frequencies were determined using the conventional Ritz method with admissible global functions consisting of a set of polynomials and a set of crack functions, including the functions presented in Equations (16) and (17). The number and orders of the polynomials and crack functions reported by Huang et al. [55] and Wang [69] are considerably larger than those of the proposed basis functions. To delay the occurrence of numerical difficulties, they developed computer codes with 128-bit precision variables and payed high computational cost. The results obtained using *N*_z_ = 3 and 4 and $N_{dx}$ × $N_{dy}$ = 50 × 25 are consistent with the results of Huang et al. up to three significant figures.

Table 2 presents the convergence of Ω for the first five modes of an FGM square plate ($\bar{m}$ = 5) and a homogeneous ($\bar{m}$ = 0) square plate (*h*/*b* = 0.1) having vertical internal central cracks with a length (*d*/*b*) of 0.5. The results in Table 2 were obtained using $(d_{m}{}_{\_in}/b,\;d_{m}{}_{\_out}/b)=$ (0.3, 0.8) and different numbers of admissible functions (*N*_z_ = 3, 4, and 5; $N_{dx}$ = $N_{dy}$ = $N_{d}$ = 15, 20, 25, and 30). The first five modal frequencies of these plates determined by the present approach decrease monotonously as *N*_z_ and $N_{d}$ increase. Again, when increasing *N*_z_ from 3 to 4, the results improve only at the fourth significant figure at most. The results obtained using *N*_z_ = 3 and 4 and $N_{d}$ = 25 and 30 converge at three significant figures.

For comparison, Table 2 also lists the results obtained with other methods apart from the proposed method. Huang et al. [54] employed the conventional Ritz method, which is highly similar to the method proposed in [55], to compute the vibration frequencies of an FGM plate without static loading. The ANSYS software by using 128,470 solid elements (C3D20 and C3D15) was employed to find the vibration frequencies of a homogeneous plate subjected to uniform compressive normal traction. The results of the present study obtained using *N*_z_ = 3 and 4 and $N_{d}$ = 25 and 30 agree well with the results obtained using other methods, with the differences being less than 0.2%.

## 4. Numerical Results

The comparisons conducted in this study verified the accuracy of the proposed solutions. Thus, the new results obtained in this study regarding the vibration frequencies of cracked FGM plates can be used as benchmark values for comparison with the results obtained using various plate theories and numerical methods. According to previous convergence studies, the following settings were considered for accurately determining the first five modal frequencies of cracked square and skewed rhombic plates: *N*_d_ = 30, *N*_z_ = 4, and $(d_{m}{}_{\_in}/b,\;d_{m}{}_{\_out}/b)=$ (0.3, 0.8), while (*N*_dx_, *N*_dy_) = (50, 25), *N*_z_ = 3, and $(d_{m}{}_{\_in}/b,\;d_{m}{}_{\_out}/b)=$ (0.5, 0.8) were adopted for rectangular plates with *a*/*b* = 2.

### 4.1. Effects of Initial Stress Components

To demonstrate the effects of different initial stress components on the frequencies, CFFF and SSSS FGM square plates with $\bar{m}$ = 0.2 and *h*/*b* = 0.1 that are subjected to static uniform uniaxial traction are considered herein. Table 4 presents the results obtained for plates with vertical side cracks, *d*/*b* = 0.5 at $c_{x}/a=$ 0.5, and uniform compressive normal stress resulting in ${\bar{\beta }}_{p}=$ 0.3. Table 5 presents the results obtained for plates with internal central vertical cracks, *d*/*b* = 0.5, and ${\bar{\beta }}_{p}=$ 0.4. The first five modal frequencies of these plates were calculated by considering three combinations of the initial stress components: (1) all $\sigma_{ij}^{\left(0\right)}$ values, (2) all the in-plane stress components only ($\sigma_{xx}^{\left(0\right)},\;\sigma_{yy}^{\left(0\right)}\;\mathrm{and}\;\sigma_{xy}^{\left(0\right)}$), and (3) only $\sigma_{xx}^{\left(0\right)}$ values. In our investigation of the CFFF boundary conditions, the differences between the frequencies obtained when considering all $\sigma_{ij}^{\left(0\right)}$ values and $\sigma_{xx}^{\left(0\right)}$ values only are less than 1.2% for the side-cracked plate and 0.5% for the internally cracked plate. These differences are larger in the case of the SSSS boundary conditions, especially for the first mode (54% and 11% for the side-cracked and internally cracked plates, respectively). However, the differences between the frequencies obtained when considering all $\sigma_{ij}^{\left(0\right)}$ values and all in-plane stress components only are 2.5% and 0.6% for the side-cracked and internally cracked plates, respectively, for the first mode.

**Figure 3:**
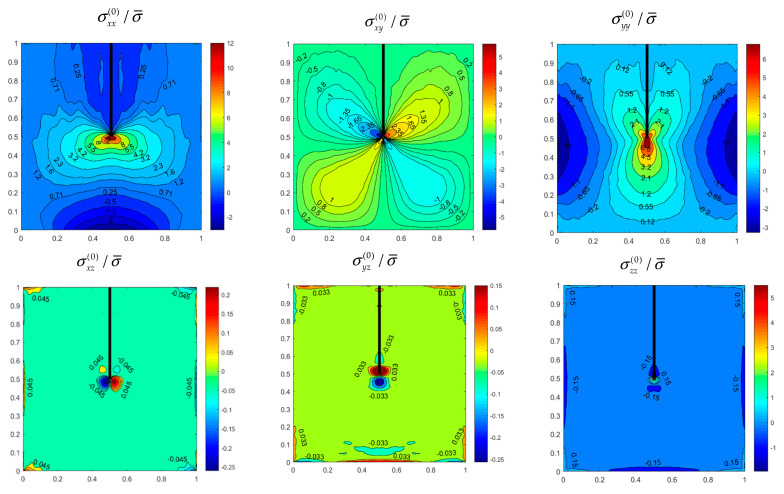
Initial Stress distributions on plane *z* = *h*/4 of an SSSS FGM plate $\left(h/b=0.1,\bar{m}=0.2\right)$ with a vertical side crack with d/b=0.5 under uniform uniaxial normal traction.

**Figure 4:**
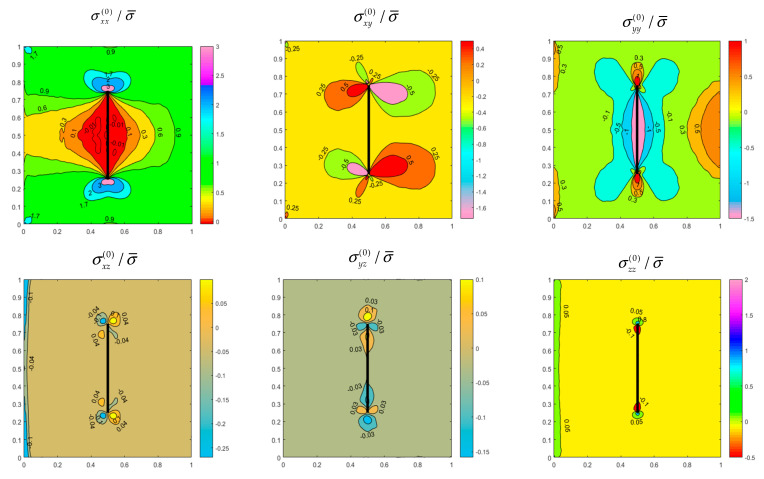
Initial Stress distributions on plane *z* = *h*/4 of a CFFF FGM plate $\left(h/b=0.1,\bar{m}=0.2\right)$ with an internal central vertical crack (d/b=0.5) under uniform uniaxial normal traction.

Figure 3 illustrates the distributions of six initial stress components in the *z* = *h*/4 plane within the SSSS FGM side-cracked plate considered in Table 4, and Figure 4 depicts the distributions for the CFFF internally cracked plate considered in Table 5. As expected, the stress components are symmetric or antisymmetric about *x*/*a* = 0.5 for the side-cracked plate, and the internally cracked plate has two symmetric planes at *y*/*b* = 0.5 and *x*/*a* = 0.5, respectively (Figure 4). Significant stress concentrations are observed near the crack tips. The stress concentration factors can be easily determined because crack functions are employed in the enriched sets of the basis functions. For the SSSS plate (Figure 3), the constraints of out-of-plane displacement on the side faces generate some out-of-plane stresses near the four side faces. Figure 3 and Figure 4 reveal that the out-of-plane stress components are considerably smaller than the in-plane stress components. Thus, the out-of-plane stress components do not significantly affect the vibration frequencies of a plate under in-plane static loading.

### 4.2. Vibration Frequencies of Side-Cracked Plates

This section describes the effects of crack configurations and static loadings on the vibration frequencies of side-cracked plates. Table 6 lists the first-five-mode nondimensional frequencies (Ω) of SSSS square plates (*h*/*b* = 0.1) with vertical side cracks at $c_{x}/a$ = 0.25; with crack lengths (*d*/*a*) of 0.1, 0.3, and 0.5; and subjected to uniform uniaxial normal traction with ${\bar{\beta }}_{p}$ = −0.5, 0, and 0.3. Table 7 lists the frequencies of CFFF rectangular plates with *a*/*b* = 1 and 2, side cracks at $c_{x}/a$ = 0.25 or 0.5, and inclination angles (β) of 90° or 135°. Negative values of ${\bar{\beta }}_{p}$ refer to tensile tractions. Notably, the same values of ${\bar{\beta }}_{p}$ for plates with different dimensions, different values of $\bar{m}$ and subjected to different boundary conditions lead to different magnitudes of prescribed tractions because the critical buckling compressive stress ${\bar{\sigma }}_{cbs}$ is included in ${\bar{\beta }}_{p}$. The required values of ${\bar{\sigma }}_{cbs}$ can be determined from Table 3.

According to Table 6 and Table 7, compressive and tensile tractions decrease and increase the vibration frequencies, respectively. The values of $R=\left|\Omega ({\bar{\beta }}_{p}\neq 0)-\Omega ({\bar{\beta }}_{p}=0)\right|/\Omega ({\bar{\beta }}_{p}=0)$ for the first mode are the largest among those of the first five modes. For example, Table 7 reveals that *R* values for the CFFF square FGM plate having a vertical side crack with a length (*d*/*b*) of 0.3 at $c_{x}/a$ = 0.25 and under compressive loading with ${\bar{\beta }}_{p}$ = 0.1 are approximately 6% for the first mode and less than 1% for the other four modes.

A crack not only reduces the stiffness of a plate but also changes the initial stress distributions of the plate. However, the results in Table 6 and Table 7 indicate that as the crack length increases, the vibration frequency significantly decreases. For example, a comparison between the frequencies of the square SSSS cracked plates with $\bar{m}$ = 0.2 (Table 6) and the intact plate reveals that a small vertical crack with a length (*d*/*a*) of 0.1 reduces the frequencies of the first four modes by less than 1% when ${\bar{\beta }}_{p}$ = 0 and 0.3 and increases these frequencies by less than 1% when ${\bar{\beta }}_{p}$ = −0.5. As the crack length increases to 0.3, the frequencies of the first four modes decrease by approximately 9%, 13%, 15%, and 35%, respectively, under static loading with ${\bar{\beta }}_{p}$ = 0.3.

For CFFF plates, Table 7 reveals that relative to the frequencies of the intact plate, the frequencies of all the first five modes except for that of the fourth mode decrease by less than 3% when a vertical crack with a length (*d*/*a*) of 0.1 appears at *x*/*a* = 0.25. Furthermore, the frequencies of the first three modes decrease by 8%–23% due to a vertical crack with a length of 0.3. An increase in the inclination angle (β) of the crack with a length of 0.3 from 90° to 135° increases the frequencies of the first four modes of the square plate. Moving a vertical crack with a length (*d*/*a*) of 0.3 from *c_x_*/*a* = 0.25 to *c_x_*/*a* = 0.5 increases the frequencies of all the first five modes of the rectangular plate with *a*/*b* = 2 except for that of the third mode.

### 4.3. Vibration Frequencies of Internally Cracked Plates

Table 8 lists the nondimensional frequencies (Ω) of the first five modes of the CFFF square and skewed rhombic (skew angle α= 15°, 30° and 45°) FGM plates ($\bar{m}=0$ and 0.2; *h*/*b* = 0.2) having central internal cracks with *d*/*b* values of 0, 0.1, 0.3 and 0.5 under uniform uniaxial stresses of magnitude ${\bar{\beta }}_{p}$ = −0.5, 0 and 0.4. The crack inclination angle (β) is 90° or 45°. Compressive loading decreases the natural frequencies of the plates, except for those of the fifth modes of the cracked skewed plates with *d*/*b* = 0.3, α= 30° and β= 45° and 90° and the second and fifth modes of the plate with *d*/*b* = 0.3, α= 45° and β= 90°. Tensile loading increases the frequencies of the plates, except for those of the fifth mode of the plate with *d*/*b* = 0.3, α= 30° and β= 90° and the second mode of the plate with *d*/*b* = 0.3, α= 45° and β= 90°.

The configurations of the crack and plate affect the vibration frequencies of the first five modes of cracked plates. A comparison of the results obtained for plates with *d*/*b* = 0.3 and ${\bar{\beta }}_{p}$ = 0 for different skew angles reveals that an increase in *α* from 0° to 15° and 30° increases the frequencies of the first four modes. Moreover, a further increase in α to 45° increases the frequencies of all the first five modes except for that of the third mode. An increase in the crack length decreases the frequencies of the square plates. The differences between the frequencies of the square plates with *d*/*b* = 0.1 and the intact plate are less than 1%, whereas the differences between the frequencies of the intact plate and the square plates with *d*/*b* = 0.3 reach approximately 7%. A decrease in the crack inclination angle (β) from 90° to 45° increases the frequencies of the first and fourth modes and decreases the frequencies of the other modes for cracked square plates with *d*/*b* = 0.3. Under the aforementioned decrease in β, the second-mode frequencies of the cracked rhombic plates with α=30° increase.

## 5. Conclusions

Proposed herein is a three-dimensional elasticity-based MLS-Ritz procedure to determine accurate vibration frequencies of preloaded cracked FGM plates. The admissible functions were constructed using the MLS technique with the proposed hybrid set of basis functions, which consists of polynomials and crack functions that properly describe the stress singular behaviors near the crack front and allow for displacement and slope discontinuities across the crack. The accuracy of the proposed solution was validated by conducting convergence studies on frequencies of the first five modes of CFFF plates with vertical cracks. The convergent results were compared against the published findings of Wang [67] and Huang et al. [53] as well as results obtained using ANSYS finite element software, and very good agreement was found.

In this study, the work done by the six initial stress components was considered in the energy formulation for free vibrations of a cracked plate under static loading. When a plate is subjected to in-plane static loading, the work done by the in-plane stress components is expected to play a more important role in affecting vibration frequencies of the plate than that done by the out-of-plane stress components. However, neglecting the work done by the out-of-plane stress components lead to a difference of more than 2% in the fundamental frequency of an SSSS side-cracked square plate with *h*/*b* = 0.1, *d*/*b* = 0.5 and $\bar{m}=0.2$ under uniform compressive normal traction of ${\bar{\beta }}_{p}$ = 0.3. The difference also depends on the boundary conditions and crack configurations under consideration.

Accurate vibration frequencies of the first five modes of cracked rectangular(*a*/*b* = 1 and 2) and skewed rhombic FGM plates subjected to uniform uniaxial static loading under the SSSS and CFFF boundary conditions were tabulated for various thickness-to-length ratios (*h*/*b* = 0.1 and 0.2), skew angles (α= 15°, 30° and 45°), crack configurations (*d*/*b* = 0.1, 0.3 and 0.5; *c_x_*/*a* = 0.25 and 0.5; β = 45°, 90° and 135°) material distributions ($\bar{m}=0$, 0.2 and 10) and loading magnitudes (${\bar{\beta }}_{p}$ = −0.5, 0, 0.1, 0.3 and 0.4). Although different crack configurations yield different distributions of static stress components, the tabulated results indicate that the first five modal frequencies of FGM plates significantly decrease as the crack length increases. This trend can be ascribed to a decrease in plate stiffness with an increase in crack length. However, relative to the frequencies of intact plates, a small crack with *d*/*b* = 0.1 results in changes of less than 1% in frequencies of an SSSS FGM square plate ($\bar{m}=0.2$, *h*/*b* = 0.1) with a vertical side crack at *c_x_*/*a* = 0.25 and a CFFF square plate ($\bar{m}=0.2$, *h*/*b* = 0.1) with a central internal vertical crack. Compressive and tensile tractions typically decrease and increase the vibration frequencies of the first five modes, respectively, but the exceptions were found in some of the second and fifth modes of CFFF skewed rhombic plates with α = 30° and 45°. The numerical results obtained in this study can be used as a benchmark for evaluating the results obtained using different approaches. More importantly, because the three-dimensional elasticity theory was employed in this study, the research results can be used to verify the suitability of different plate theories for representing the vibration of cracked plates.

## Figures and Tables

**Table 1:** Convergence of frequency parameters Ω for CFFF FGM ($\bar{m}=0.2$) rectangular. Plates (a/b = 2, *h*/*b* = 0.1) having vertical side cracks ($c_{x}/a=0.5$, *d/a* = 0.5).

${\bar{\beta }}_{p}$	Mode	*N*_z_	$N_{dx}$ × $N_{dy}$				Wang [69]
			25 × 25	40 × 20	30 × 30	50 × 25	
0	1	2	0.2271	0.2269	0.2270	0.2269	0.227
		3	0.2268	0.2267	0.2268	0.2266	
		4	0.2268	0.2267	0.2268	0.2266	
	2	2	0.8422	0.8415	0.8421	0.8410	0.838
		3	0.8396	0.8389	0.8395	0.8384	
		4	0.8396	0.8388	0.8395	0.8384	
	3	2	1.263	1.261	1.262	1.260	1.255
		3	1.259	1.258	1.259	1.257	
		4	1.259	1.257	1.259	1.256	
	4	2	1.532 *	1.531 *	1.532 *	1.531 *	1.530
		3	1.532 *	1.531 *	1.532 *	1.530 *	
		4	1.532 *	1.531 *	1.532 *	1.530 *	
	5	2	2.745	2.742	2.743	2.741	2.726
		3	2.731	2.728	2.729	2.727	
		4	2.731	2.728	2.729	2.727	
0.3	1	2	0.1838	0.1836	0.1837	0.1835	/
		3	0.1834	0.1833	0.1834	0.1832	
		4	0.1834	0.1833	0.1834	0.1831	
	2	2	0.8400	0.8392	0.8399	0.8388	/
		3	0.8374	0.8366	0.8373	0.8362	
		4	0.8373	0.8366	0.8373	0.8362	
	3	2	1.222	1.220	1.222	1.219	/
		3	1.218	1.217	1.218	1.216	
		4	1.218	1.217	1.218	1.215	
	4	2	1.525 *	1.524 *	1.525 *	1.524 *	/
		3	1.525 *	1.524 *	1.525 *	1.524 *	
		4	1.525 *	1.524 *	1.525 *	1.524 *	
	5	2	2.734	2.731	2.732	2.729	/
		3	2.720	2.717	2.718	2.716	
		4	2.720	2.717	2.718	2.715	

Note: / denotes no data available.

**Table 2:** Convergence of frequency parameters Ω for CFFF square plates (*h*/*b* = 0.1) having vertical internal central cracks (*d/a* = 0.5).

$\bar{m}$	${\bar{\beta }}_{p}$	Mode	*N*_z_	$N_{d}$				Different Approaches
				15	20	25	30	
5	0	1	2	0.6463	0.6456	0.6451	0.6450	(0.6423)
			3	0.6437	0.6430	0.6425	0.6425	
			4	0.6436	0.6429	0.6424	0.6423	
		2	2	1.582	1.579	1.579	1.578	(1.561)
			3	1.565	1.562	1.562	1.561	
			4	1.564	1.562	1.561	1.561	
		3	2	3.427	3.418	3.410	3.409	(3.365)
			3	3.386	3.377	3.371	3.370	
			4	3.385	3.376	3.369	3.368	
		4	2	3.965 *	3.962 *	3.961 *	3.961 *	(3.960)
			3	3.964 *	3.961 *	3.960 *	3.959 *	
			4	3.964 *	3.961 *	3.959 *	3.959 *	
		5	2	4.856	4.853	4.851	4.851	(4.808)
			3	4.815	4.813	4.811	4.811	
			4	4.814	4.812	4.810	4.809	
0	0.1	1	2	0.9257	0.9246	0.9236	0.9235	[0.9210]
			3	0.9232	0.9221	0.9212	0.9210	
			4	0.9231	0.9220	0.9211	0.9209	
		2	2	2.400	2.395	2.394	2.393	[2.378]
			3	2.384	2.380	2.378	2.378	
			4	2.384	2.379	2.378	2.378	
		3	2	5.192	5.176	5.164	5.162	[5.129]
			3	5.155	5.140	5.129	5.127	
			4	5.154	5.139	5.128	5.126	
		4	2	6.095 *	6.091 *	6.089 *	6.089 *	[6.081]
			3	6.095 *	6.091 *	6.088 *	6.088 *	
			4	6.094 *	6.090 *	6.087 *	6.087 *	
		5	2	7.436	7.433	7.429	7.429	[7.392]
			3	7.401	7.397	7.394	7.394	
			4	7.401	7.397	7.394	7.393	

Note: ( ) denotes results of Huang et al. [54]; [ ] denotes results obtained from ANSYS.

**Table 3:** Nondimensional critical buckling loads for intact plates.

*h/b*	B.C.	*a/b*	α	$\bar{m}$	${\bar{N}}_{cbs}$
0.1	CFFF	1	0°	0	0.2377
				0.2	1.059
		2		0.2	0.2610
	SSSS	1		0	3.376
				0.2	15.09
				10	5.349
		2		0.2	15.42
0.2	CFFF	1	0°	0	0.2308
				0.2	1.029
			15°	0.2	1.029
			30°		1.021
			45°		0.9892

**Table 4:** Non-dimension frequencies (Ω) of CFFF and SSSS side-cracked FGM square plates. (*h*/*b* = 0.1, $\bar{m}$ = 0.2 and d/b = 0.5) with considering different initial stress components.

B.C.	${\bar{\beta }}_{p}$	$\sigma_{ij}^{\left(0\right)}$	Mode				
			1	2	3	4	5
CFFF	0.3	All $\sigma_{ij}^{\left(0\right)}$	0.6288	1.598	3.643 *	4.022	5.566
		$\sigma_{xx}^{\left(0\right)},\sigma_{xy}^{\left(0\right)},\sigma_{yy}^{\left(0\right)}$	0.6288	1.598	3.643 *	4.022	5.566
		$\sigma_{xx}^{\left(0\right)}$	0.6364	1.585	3.646*	4.000	5.547
SSSS		All $\sigma_{ij}^{\left(0\right)}$	2.280	5.560 *	8.054	11.16	14.72
		$\sigma_{xx}^{\left(0\right)},\sigma_{xy}^{\left(0\right)},\sigma_{yy}^{\left(0\right)}$	2.222	5.540 *	7.957	11.12	14.68
		$\sigma_{xx}^{\left(0\right)}$	3.499	5.671 *	8.554	11.82	14.63

**Table 5:** Non-dimension frequencies (Ω) of CFFF and SSSS FGM square plates with internal central vertical cracks (*h*/*b* = 0.1, $\bar{m}$ = 0.2 and d/b = 0.5) and considering different initial stress components.

B.C.	${\bar{\beta }}_{p}$	$\sigma_{ij}^{\left(0\right)}$	Mode				
			1	2	3	4	5
CFFF	0.4	All $\sigma_{ij}^{\left(0\right)}$	0.657	2.157	4.596	5.784 *	6.866
		$\sigma_{xx}^{\left(0\right)},\sigma_{xy}^{\left(0\right)},\sigma_{yy}^{\left(0\right)}$	0.657	2.158	4.597	5.785 *	6.866
		$\sigma_{xx}^{\left(0\right)}$	0.660	2.158	4.601	5.785 *	6.867
SSSS		All $\sigma_{ij}^{\left(0\right)}$	3.747	8.934	12.13	16.47 *	17.09
		$\sigma_{xx}^{\left(0\right)},\sigma_{xy}^{\left(0\right)},\sigma_{yy}^{\left(0\right)}$	3.769	8.930	12.15	16.46 *	17.08
		$\sigma_{xx}^{\left(0\right)}$	3.318	8.921	11.89	16.52 *	17.07

**Table 6:** Nondimensional frequencies (Ω) of SSSS FGM square plates (*h*/*b* = 0.1) with vertical side cracks at $c_{x}/a$ = 0.25.

$\bar{m}$	d/b	${\bar{\beta }}_{p}$	Mode				
			1	2	3	4	5
0	0	−0.5	6.798	14.06	15.58	21.94	24.53 *
		0	5.552	13.49	13.49	20.50	24.33 *
		0.3	4.645	12.06	13.13	19.59	23.92
	0.3	−0.5	7.044	12.05 *	14.19	15.64	21.52
		0	5.474	11.01 *	12.98	13.44	19.75
		0.3	4.208	10.26 *	11.10	12.82	18.50
0.2	0	−0.5	6.328	13.10	14.52	20.48	23.36 *
		0	5.167	12.57	12.57	19.14	23.18 *
		0.3	4.322	11.24	12.24	18.29	22.36
	0.1	−0.5	6.344	13.13 *	14.54	20.51	22.87
		0	5.161	12.55 *	12.57	19.11	22.33
		0.3	4.297	11.21 *	12.21	18.22	21.91
	0.3	−0.5	6.556	11.45 *	13.23	14.57	20.10
		0	5.096	10.49 *	12.10	12.53	18.46
		0.3	3.919	9.813 *	10.36	11.95	17.30
	0.5	−0.5	6.309 *	6.767	12.58	14.01	17.04 *
		0	4.843	5.436 *	10.89	11.97	15.31
		0.3	2.438	4.685 *	7.938	11.23	12.46
10	0.3	−0.5	4.416	7.204 *	8.823	9.735	13.26
		0	3.430	6.516 *	8.050	8.344	12.13
		0.3	2.630	6.005 *	6.844	7.948	11.31

**Table 7:** Nondimensional frequencies (Ω) of CFFF FGM plates (*h*/*b* = 0.1) with side cracks.

*a/b*	$\bar{m}$	$c_{x}/a$	*d/b*	β	${\bar{\beta }}_{p}$	Mode				
						1	2	3	4	5
1	0	0.25	0.3	90°	−0.5	1.152	2.142	4.816 *	5.405	7.620
					0	0.9240	2.096	4.777 *	5.216	7.512
					0.3	0.7423	2.067	4.753 *	5.093	7.435
	0.2		0		−0.5	1.163	2.337	5.907	6.308 *	7.223
					0	0.9667	2.273	5.676	6.286 *	7.190
					0.3	0.8186	2.234	5.530	6.272 *	7.172
			0.1	90°	−0.5	1.149	2.298	5.845	6.023 *	7.198
					0	0.9497	2.237	5.620	5.999 *	7.164
					0.3	0.7984	2.200	5.477	5.984 *	7.145
			0.3	90°	−0.5	1.071	1.993	4.587 *	5.033	7.089
					0	0.8585	1.950	4.551 *	4.858	6.989
					0.1	0.8071	1.941	4.544 *	4.821	6.967
					0.3	0.6899	1.924	4.530 *	4.744	6.919
				135°	−0.5	1.136	2.135	5.034	5.727 *	6.969
					0	0.9301	2.085	4.851	5.699 *	6.914
					0.3	0.7703	2.054	4.732	5.681 *	6.878
			0.5	90°	−0.5	0.9337	1.560	2.962 *	4.027	6.926
					0	0.7164	1.523	2.909 *	3.876	6.900
					0.3	0.5192	1.502	2.877 *	3.777	6.802
2	0.2	0.25	0.3	90°	−0.5	0.2773	0.9274	1.513	1.609 *	3.077
					0	0.2256	0.9217	1.453	1.602 *	3.056
					0.3	0.1855	0.9181	1.416	1.597 *	3.044
				135°	−0.5	0.2847	0.9612	1.508	1.882 *	3.130
					0	0.2342	0.9546	1.448	1.876 *	3.108
					0.3	0.1955	0.9506	1.411	1.872 *	3.095
		0.5	0.3	90°	−0.5	0.2868	0.9478	1.447	1.899 *	3.098
					0	0.2359	0.9419	1.384	1.892 *	3.079
					0.3	0.1966	0.9384	1.345	1.888 *	3.068

**Table 8:** Nondimensional frequencies (Ω) of CFFF square and skewed rhombic FGM plates (*h*/*b* = 0.2) with central internal cracks.

α	$\bar{m}$	*d*/*b*	β	${\bar{\beta }}_{p}$	Mode				
					1	2	3	4	5
0°	0	0.3	90°	−0.5	1.203	2.274	3.268 *	5.289	6.710
				0	0.9890	2.194	3.218 *	5.009	6.686
				0.4	0.7545	2.127	3.177 *	4.771	6.668
	0.2	0		−0.5	1.133	2.147	3.192 *	5.241	6.405
				0	0.9442	2.083	3.149 *	5.010	6.372
				0.4	0.7451	2.029	3.114 *	4.813	6.348
		0.1		−0.5	1.131	2.143	3.184 *	5.203	6.381
				0	0.9412	2.077	3.140 *	4.967	6.351
				0.4	0.7397	2.022	3.105 *	4.766	6.328
		0.3		−0.5	1.119	2.122	3.112 *	4.943	6.260
				0	0.9198	2.048	3.066 *	4.683	6.239
				0.4	0.7017	1.986	3.029 *	4.463	6.222
0°	0.2	0.3	45°	−0.5	1.126	2.106	3.110 *	5.122	6.083
				0	0.9323	2.035	3.064 *	4.874	6.056
				0.4	0.7247	1.976	3.027 *	4.664	6.034
		0.5	90°	−0.5	1.094	2.103	2.960 *	4.510	5.901 *
				0	0.8772	2.013	2.910 *	4.234	5.873 *
				0.4	0.6236	1.937	2.868 *	3.996	5.849 *
15°		0.3		−0.5	1.150	2.151	3.126 *	5.109	6.123
				0	0.9452	2.090	3.077 *	4.827	6.085
				0.4	0.7211	2.039	3.037 *	4.585	6.052
30°				−0.5	1.241	2.271	3.149 *	5.619	6.038
				0	1.021	2.249	3.087 *	5.249	6.045
				0.4	0.7803	2.227	3.037 *	4.916	6.061
			45°	−0.5	1.259	2.271	3.147 *	5.821	5.937
				0	1.049	2.251	3.086 *	5.499	5.921
				0.4	0.8253	2.231	3.036 *	5.171	5.955
45°			90°	−0.5	1.372	2.607	3.090 *	6.177	6.541
				0	1.142	2.644	3.004 *	5.756	6.539
				0.4	0.8786	2.669	2.933 *	5.272	6.613

## Appendix A

$$\begin{array}{l} f_{m}^{1}=\int_{s}\phi_{1i}(-t_{x}\cos\beta -t_{y}\sin\beta )ds\int_{-h/2}^{h/2}z^{k}dz;f_{m}^{2}=\int_{s}\phi_{2i}(t_{x}\sin\beta -t_{y}\cos\beta )ds\int_{-h/2}^{h/2}z^{k}dz;\;f_{m}^{3}=\int_{s}\phi_{3i}t_{z}ds\int_{-h/2}^{h/2}z^{k}dz;\\ \begin{array}{ll} {Κ}_{mn}^{11} & =\iint_{A}[2\phi_{1i,x}\phi_{1j,x}\cos^{2}\beta +2\phi_{1i,y}\phi_{1j,y}\sin^{2}\beta +(\phi_{1i,y}\cos\beta +\phi_{1i,x}\sin\beta )(\phi_{1j,y}\cos\beta +\phi_{1j,x}\sin\beta )]dA\\ & \int_{-h/2}^{h/2}G(z)z^{k+l}dz+\iint_{A}kl\phi_{1i}\phi_{1j}dA\int_{-h/2}^{h/2}G(z)z^{k+l-2}dz\\ & +\iint_{A}\left(\phi_{1i,x}\cos\beta +\phi_{1i,y}\sin\beta )(\phi_{1j,x}\cos\beta +\phi_{1j,y}\sin\beta \right)dA\int_{-h/2}^{h/2}\lambda (z)z^{k+l}dz; \end{array}\\ \begin{array}{ll} {Κ}_{mn}^{22} & =\iint_{A}[2\phi_{2i,x}\phi_{2j,x}\sin^{2}\beta +2\phi_{2i,y}\phi_{2j,y}\cos^{2}\beta +(\phi_{2i,y}\sin\beta -\phi_{2i,x}\cos\beta )(\phi_{2j,y}\sin\beta -\phi_{2j,x}\cos\beta )]dA\\ & \int_{-h/2}^{h/2}G(z)z^{k+l}dz+\iint_{A}kl\phi_{2i}\phi_{2j}dA\int_{-h/2}^{h/2}G(z)z^{k+l-2}dz\\ & +\iint_{A}\left(-\phi_{2i,y}\cos\beta +\phi_{2i,x}\sin\beta )(-\phi_{2j,y}\cos\beta +\phi_{2j,x}\sin\beta \right)dA\int_{-h/2}^{h/2}\lambda (z)z^{k+l}dz; \end{array}\\ \begin{array}{ll} {Κ}_{mn}^{33}= & \iint_{A}\left(\phi_{3i,x}\phi_{3j,x}+\phi_{3i,y}\phi_{3j,y}\right)dA\int_{-h/2}^{h/2}G(z)z^{k+l}dz+\iint_{A}2kl\left(\phi_{3i}\phi_{3j}\right)dA\int_{-h/2}^{h/2}G(z)z^{k+l-2}dz\\ & +\iint_{A}kl\phi_{3i}\phi_{3j}dA\int_{-h/2}^{h/2}\lambda (z)z^{k+l-2}dz; \end{array}\\ \begin{array}{ll} {Κ}_{mn}^{12} & =\iint_{A}[\left(\phi_{1i,y}\cos\beta +\phi_{1i,x}\sin\beta )(\phi_{2j,x}\cos\beta -\phi_{2j,y}\sin\beta \right)+2\cos\beta \sin\beta (-\phi_{1i,x}\phi_{2j,x}+\phi_{1i,y}\phi_{2j,y})]dA\\ & \int_{-h/2}^{h/2}G(z)z^{k+l}dz+\iint_{A}\left(\phi_{1i,x}\cos\beta +\phi_{1i,y}\sin\beta \right)(\phi_{2j,y}\cos\beta -\phi_{2j,x}\sin\beta )dA\int_{-h/2}^{h/2}\lambda (z)z^{k+l}dz; \end{array}\\ \begin{array}{ll} {Κ}_{mn}^{13}= & \iint_{A}k\left(-\phi_{3j,x}\cos\beta -\phi_{3j,y}\sin\beta \right)\phi_{1i}dA\int_{-h/2}^{h/2}G(z)z^{k+l-1}dz\\ & +\iint_{A}l\left(-\phi_{1i,x}\cos\beta -\phi_{1i,y}\sin\beta \right)\;\phi_{3j}dA\int_{-h/2}^{h/2}\lambda (z)z^{k+l-1}dz; \end{array}\\ \begin{array}{ll} {Κ}_{mn}^{23}= & \iint_{A}k\left(-\phi_{3j,y}\cos\beta +\phi_{3j,x}\sin\beta \right)\phi_{2i}dA\int_{-h/2}^{h/2}G(z)z^{k+l-1}dz\\ & +\iint_{A}l\left(-\phi_{2i,y}\cos\beta +\phi_{2i,x}\sin\beta \right)\;\phi_{3j}dA\int_{-h/2}^{h/2}\lambda (z)z^{k+l-1}dz; \end{array}\\ \begin{array}{ll} K_{gmn}^{11} & =\iint_{A}\left[\sigma_{xx}^{\left(0\right)}\phi_{1i,x}\phi_{1j,x}+\sigma_{yy}^{\left(0\right)}\phi_{1i,x}\phi_{1j,x}+\sigma_{xy}^{\left(0\right)}(\phi_{1i,x}\phi_{1j,y}+\phi_{1i,y}\phi_{1j,x})\right]dA\int_{-h/2}^{h/2}z^{k+l}dz\\ & +\iint_{A}\left[\sigma_{xz}^{\left(0\right)}(l\phi_{1i,x}\phi_{1j}+k\phi_{1i}\phi_{1j,x})+\sigma_{yz}^{\left(0\right)}(l\phi_{1i,y}\phi_{1j}+k\phi_{1i}\phi_{1j,y})\right]dA\int_{-h/2}^{h/2}z^{k+l-1}dz+\iint_{A}\sigma_{zz}^{\left(0\right)}kl\phi_{1i}\phi_{1j}dA\int_{-h/2}^{h/2}z^{k+l-2}dz; \end{array}\\ \begin{array}{ll} K_{gmn}^{22} & =\iint_{A}\left[\sigma_{xx}^{\left(0\right)}\phi_{2i,x}\phi_{2j,x}+\sigma_{yy}^{\left(0\right)}\phi_{2i,x}\phi_{2j,x}+\sigma_{xy}^{\left(0\right)}(\phi_{2i,x}\phi_{2j,y}+\phi_{2i,y}\phi_{2j,x})\right]dA\int_{-h/2}^{h/2}z^{k+l}dz\\ & +\iint_{A}\left[\sigma_{xz}^{\left(0\right)}(l\phi_{2i,x}\phi_{2j}+k\phi_{2i}\phi_{2j,x})+\sigma_{yz}^{\left(0\right)}(l\phi_{2i,y}\phi_{2j}+k\phi_{2i}\phi_{2j,y})\right]dA\;\int_{-h/2}^{h/2}z^{k+l-1}dz+\iint_{A}\sigma_{zz}^{\left(0\right)}kl\phi_{2i}\phi_{2j}dA\int_{-h/2}^{h/2}z^{k+l-2}dz; \end{array}\\ \begin{array}{ll} K_{gmn}^{33} & =\iint_{A}\left[\sigma_{xx}^{\left(0\right)}\phi_{3i,x}\phi_{3j,x}+\sigma_{yy}^{\left(0\right)}\phi_{3i,x}\phi_{3j,x}+\sigma_{xy}^{\left(0\right)}(\phi_{3i,x}\phi_{3j,y}+\phi_{3i,y}\phi_{3j,x})\right]dA\int_{-h/2}^{h/2}z^{k+l}dz\\ & +\iint_{A}\left[\sigma_{xz}^{\left(0\right)}(l\phi_{3i,x}\phi_{3j}+k\phi_{3i}\phi_{3j,x})+\sigma_{yz}^{\left(0\right)}(l\phi_{3i,y}\phi_{3j}+k\phi_{3i}\phi_{3j,y})\right]dA\int_{-h/2}^{h/2}z^{k+l-1}dz+\iint_{A}\sigma_{zz}^{\left(0\right)}kl\phi_{3i}\phi_{3j}dA\int_{-h/2}^{h/2}z^{k+l-2}dz; \end{array}\\ M_{mn}^{11}=\iint_{A}\phi_{1i}\phi_{1j}dA\int_{-h/2}^{h/2}\rho (z)z^{k+l}dz;M_{mn}^{22}=\iint_{A}\phi_{2i}\phi_{2j}dA\int_{-h/2}^{h/2}\rho (z)z^{k+l}dz;M_{mn}^{33}=\iint_{A}\phi_{3i}\phi_{3j}dA\int_{-h/2}^{h/2}\rho (z)z^{k+l}dz; \end{array}$$

where $\lambda (z)=\frac{\nu (z)E(z)}{\left(1+\nu (z))(1-2\nu (z)\right)}$, $G(z)=\frac{E(z)}{2(1+\nu (z))}$, $m=i+kN_{p}\;\mathrm{and}\;n=j+lN_{p}$.
